# Cauda equina syndrome in an ovarian malignant‐mixed müllerian tumor with leptomeningeal spread

**DOI:** 10.1002/ccr3.2472

**Published:** 2019-10-22

**Authors:** Joshua D. Bernstock, Stuart Ostby, Brandon Fox, Houman Sotoudeh, Andrew Janssen, Yun Jee Kang, Jason Chen, Veeranjaneyulu Prattipati, Galal Elsayed, Gustavo Chagoya, Daisuke Yamashita, Gregory K. Friedman, Burt Nabors, Warner K. Huh, Mina Lobbous

**Affiliations:** ^1^ Department of Neurosurgery Brigham and Women's Hospital Harvard University Boston MA USA; ^2^ Division of Gynecologic Oncology University of Alabama at Birmingham Birmingham AL USA; ^3^ School of Medicine University of Alabama at Birmingham Birmingham AL USA; ^4^ Department of Radiology, Neuroradiology Section University of Alabama at Birmingham Birmingham AL USA; ^5^ Harvard Medical School Boston MA USA; ^6^ Medical Scientist Training Program University of California Los Angeles CA USA; ^7^ Department of Neurosurgery University of Alabama at Birmingham Birmingham AL USA; ^8^ Division of Pediatric Hematology and Oncology Department of Pediatrics University of Alabama at Birmingham Birmingham AL USA; ^9^ Department of Neurology University of Alabama at Birmingham Birmingham AL USA

**Keywords:** cauda equina syndrome, leptomeningeal carcinomatosis, metastasis, ovarian carcinosarcoma, ovarian malignant‐mixed müllerian tumor

## Abstract

Leptomeningeal metastasis is extremely rare in patients with ovarian cancer, but should be considered in patients presenting with neurologic deficits such as cauda equine syndrome. Given its poor prognosis and lack of data currently on management, additional studies are needed to optimize treatment regimens and improve outcomes.

## Graphical abstract

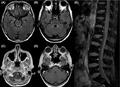

## 1 INTRODUCTION

Ovarian cancer is the most common cause of mortality among women with gynecologic malignancies and the fifth leading cause of death in all women.^1^, ^2^, ^3^ Ovarian cancer is comprised of a diverse group of tumors that arise from multiple cell types and anatomical sites.^4^ Epithelial‐derived tumors represent ~90% of ovarian cancers and are classified based upon cellular histology as well as clinical and pathologic profiling.^3^, ^5^ The majority of epithelial ovarian cancer cases are diagnosed at stage III or IV, and 5‐year overall survival is less than 50%.^3^ Rarely, primary ovarian cancer can be made up of both malignant epithelial and mesenchymal components, and these tumors, termed ovarian malignant‐mixed müllerian tumor (OMMMT), or alternately known as ovarian carcinosarcomas, are highly malignant and carry a poor prognosis.^6^

Central nervous system (CNS) metastasis is uncommon in ovarian cancer and occurs in approximately 1% of patients.^7^, ^8^ Leptomeningeal metastatic disease (LMD) is a rare pattern of CNS metastasis most commonly involving the basal cisterns, posterior fossa, and cauda equina.^9^ While LMD may present with focal symptoms, most frequently patients experience nonspecific symptoms including confusion, nausea, and headache. When the cauda equina is widely affected, cauda equina syndrome may result and be the first presenting symptom indicating CNS metastasis.^10^, ^11^

Herein, we describe a case of OMMMT with LMD presenting with cauda equina syndrome and review the clinical course, neuroradiological findings, and pertinent literature.

## 2 CASE

A 62‐year‐old postmenopausal female who was otherwise healthy presented with an adnexal mass measuring 11 × 8 × 9cm and recurrent fevers. Computerized tomography (CT) imaging demonstrated a right adnexal mass with ascites, a separate cystic pelvic mass, and intraparenchymal liver lesions concerning for advanced ovarian cancer. Initial CA125 was 1480, and her albumin was 3.1. Given her diminished nutritional status and normal performance status, laparoscopic biopsies were performed with a preliminary diagnosis of metastatic high‐grade serous carcinoma. She underwent genetic counseling and testing, which was negative for Lynch syndrome; she declined BRCA 1 and 2 testing. Next‐generation sequencing of the tumor identified somatic mutations in *FGFR1*, *TP53*, and *ZNF703*.

Neoadjuvant chemotherapy followed by interval debulking surgery was recommended and the patient participated in clinical trial GOG 3005 (NCT02470585). Following randomization, she was treated in the control arm with paclitaxel and carboplatin without veliparib. While receiving neoadjuvant chemotherapy, she suffered a deep venous thrombosis (DVT) and underwent anticoagulation and placement of an IVC filter. After cycle four, she underwent radical cytoreduction with exploratory laparotomy, hysterectomy, bilateral salpingo‐oophorectomy with removal of bilateral adnexal masses, and pelvic peritonectomy, and was found to have miliary carcinoma of small bowel mesentery, right hemidiaphragm, and gastrocolic ligament. Estimated resection was subtotal with removal of ~90% percent of visualized disease. After an uncomplicated postoperative course, her IVC filter was removed. After cycle five, she had a biochemical increase in CA125 leading to discontinuation in the clinical trial due to possible progression of disease.

The patient was transitioned to a nonplatinum doublet consisting of doxorubicin and bevacizumab. She displayed symptomatic improvement and an objective response by RECIST criteria despite an increasing CA125 after eight cycles. Due to the discrepancy, positron‐emission tomography (PET)/CT was utilized and demonstrated fluorodeoxyglucose ([^18^F]FDG) activity in the right humerus and bilateral femurs consistent with metastatic disease for which the patient was asymptomatic. Given partial response to retesting with platinum, she transitioned to rucaparib for maintenance therapy. This was continued until developing progression with carcinomatosis after three months.

Operative laparoscopy was repeated with biopsies and followed by intraperitoneal cisplatin therapy for six cycles. Next‐generation sequencing of repeat tumor biopsies demonstrated microsatellite stability without new targetable therapies. Following her sixth cycle of intraperitoneal cisplatin, she had progressive hepatic disease and a new right axillary mass. In the absence of alternative therapies, she sought use of immunotherapy with pembrolizumab.

**Figure 1:**
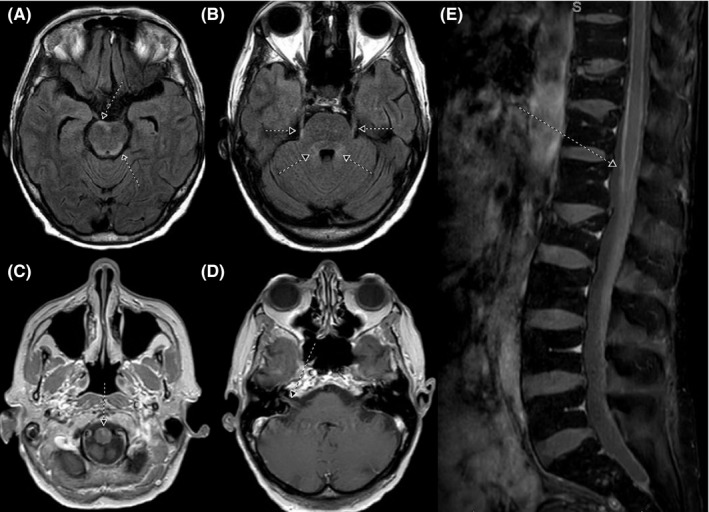
Axial FLAIR sequence shows mild FLAIR signal hyperintensity within the ventral aspect of pons, dorsal brainstem, along the trigeminal nerves, and surrounding the fourth ventricle (arrows, A and B). Axial postcontrast T1 sequence shows leptomeningeal enhancement around the upper cervical cord and enhancement along the right 7th and 8th nerves (arrows, C and D). Postcontrast sagittal fat suppression sequence shows mild leptomeningeal enhancement around the conus medullaris (arrow, E) with an incidental old compression deformity of the L2 vertebral body

After 18 months from original diagnosis, she developed difficulty voiding and was concerned about a possible urinary tract infection. Her intraperitoneal port was removed, and she started on pembrolizumab. Following cycle one she was directly admitted to the hospital from home due to acute urinary retention, gait imbalance, headache, vision changes, and a CT scan which demonstrated a lesion in her right temporal lobe. Her urinary retention was relieved via the placement of a catheter, and she subsequently underwent magnetic resonance imaging (MRI) of the brain and spine which demonstrated leptomeningeal enhancement of the ventral surface of the pons, right cerebral hemisphere, and the spinal cord (ie, from thoracic spine to the cauda equine; Figure 1). Of note, there was also enhancement of the trigeminal, facial, and vestibulocochlear cranial nerves.

The patient was continued on pembrolizumab and additionally treated with systemic high‐dose methotrexate. Cerebrospinal fluid (CSF) analysis demonstrated increased leukocytes, macrophages, and protein with decreased glucose, with an opening pressure of 22 cm of H_2_O; anti‐Yo antibodies were negative, and the CSF cytology was positive for malignancy. She demonstrated no response to high‐dose intrathecal (IT) methotrexate without change in cauda equina symptoms and ultimately decided to pursue hospice care.

## 3 DISCUSSION

Ovarian malignant‐mixed müllerian tumor, also referred to as carcinosarcoma of the ovary, is a very rare and aggressive histologic subtype typically presenting in older patients and at more advanced stages.^12^ OMMMT is a neoplasm comprised of epithelial and mesenchymal tissues of müllerian (paramesonephric duct) origin. The complete pathobiology/pathogenesis of OMMMT remains unclear with immunologic studies supporting malignant elements as originating from a common epithelial stem cell as opposed to convergence of two separate malignancies.^13^ As a rare subtype of ovarian cancer which is partly of epithelial origin, the International Federation of Gynecology and Obstetrics (FIGO) staging system is used^14^ and platinum‐based chemotherapy is recommended despite lower response rates.^15^, ^16^, ^17^ Overall survival is significantly lower with median survival of 4‐14 months.^18^, ^19^ Unlike uterine carcinosarcoma, lung, and other distant metastases are still uncommon, occurring in ~5% of patients diagnosed with OMMMT.^18^, ^20^

### 3.1 Leptomeningeal disease in ovarian cancers

The common sites for metastasis from epithelial ovarian cancer include the abdomen, liver, and lungs; the metastases are typically spread by seeding of the peritoneal cavity, and only occasionally hematogenously. CNS metastasis in ovarian cancer is reported in approximately 1% of cases and typically localizes to the parenchyma; once in the CNS, metastatic disease can spread by direct seeding through the CSF. Leptomeningeal disease (LMD) is exceedingly rare.^7^, ^21^, ^22^ In a case series of 23 patients with CNS metastases from primary ovarian carcinoma,^23^ only 1 patient was found to have LMD. A 2005 retrospective study of 1,450 patients with primary ovarian malignancies found CNS metastases in only 17 patients (ie, 1.17%) without a case of meningeal involvement.

The molecular understanding of OMMMT through next‐generation whole‐exome sequencing 24 has identified serous adenomatous components and shared alterations in *TP53* within high‐grade serous tumors.^25^ Generally, epithelial ovarian cancers are capable of inducing strong immune responses, highlighting the potential use of PD‐1 and PD‐L1 inhibitors in ovarian cancer 26, 27, 28, 29 with overall response rates ranging from 9.7% to 25%.

Leptomeningeal metastatic disease usually presents with symptoms of increased intracranial pressure, manifesting clinically as headaches, nausea, and/or vomiting; however, it may also cause focal neurologic deficits such as cranial nerve deficits, cerebellar symptoms, and/or cauda equine, as in our patient.^30^, ^31^ Gadolinium‐enhanced MRI of the neuroaxis may show increased enhancement in the leptomeninges, dura, cranial nerves, or cauda equina especially helpful in patients raising high suspicion of CNS metastasis and LMD. The gold standard in diagnosing LMD is the detection of malignant cells on CSF cytology; however, false‐negative results may affect half of patients on initial lumbar puncture.^32^ Imaging should be performed prior to lumbar puncture whenever possible as lumbar puncture may occasionally produce false‐positive signal intensity on gadolinium MRI.^30^ The standard treatment of LMD, regardless of the tumor histology, includes palliative CSF diversion for hydrocephalus, radiation therapy, and either IT or systemic chemotherapy.^33^ Median survival for LMD is generally from 8 to 16 weeks with standard interventions.^34^, ^35^, ^36^, ^37^

Very limited information exists regarding LMD in patients with epithelial ovarian carcinoma. Once LMD has developed, prognosis is extremely poor with median survival of 60 days.^38^, ^39^, ^40^, ^41^, ^42^, ^43^, ^44^, ^45^, ^46^, ^47^ Of note, new treatment regimens for ovarian cancers have increased survival time leading to new opportunity in treating CNS metastases.^48^ Given the extreme rarity of LMD in ovarian cancers, there is no general consensus on the best treatment approach. Historically, treatment has focused on an IT chemotherapy and most commonly methotrexate has been used with or without whole‐brain radiotherapy (WBRT).^44^ Additional chemotherapeutic agents with appropriate CNS penetration include cisplatin, systemic methotrexate, IT thiotepa, and IT topotecan.^21^ High‐dose systemic methotrexate (8 g/m^2^) was studied in a prospective, nonrandomized cohort compared to IT methotrexate for treatment of LMD and favored systemic administration (survival 13.8 versus 2.3 months, *P* = .003). Most recently, systemic therapy with agents producing adequate CSF concentrations has been promoted as preferred therapy for LMD.^49^, ^50^, ^51^ Despite such advances prognosis remains dismal and more work is therefore required to advance treatment for patients in need.

## 4 CONCLUSION

Ovarian malignant‐mixed müllerian tumor with LMD presenting is extremely rare and remains clinically difficult to manage. Further studies and clinical trials examining novel therapeutics as well as combinations of therapies are needed to provide improvements in overall survival for ovarian patients with LMD.

## CONFLICT OF INTEREST

None declared.

## AUTHOR CONTRIBUTIONS

All authors participated in the clinical care of the patient and/or the drafting/revising of the manuscript.
